# Coronal Plane Screening of Lower Limb Deformity

**DOI:** 10.5704/MOJ.2207.024

**Published:** 2022-07

**Authors:** RY Kow, CL Low, MN Yusof

**Affiliations:** 1 Department of Orthopaedics, Traumatology and Rehabilitation, International Islamic University Malaysia, Kuantan, Malaysia; 2 Department of Radiology, Sultan Ahmad Shah Medical Centre @IIUM, Kuantan, Malaysia

Dear editor,

Lower limb deformity is defined as deviation of the normal alignment or orientation of the affected limb^1,2^. Pre-operative planning for deformity correction involves identification of the cause of deformity, be it from the bone (femur or tibia), the joint (hip, knee or ankle) or both the bone and joint involvement. Nowadays, malalignment and malorientation test, described by Paley et al, is routinely being used in preoperative planning to delineate and identify the pathological site^1,2^. Nevertheless, this technique is relatively difficult to comprehend especially by inexperienced personnel. This is due to multiple seemingly identical measurements such as lateral proximal femur angle (LPFA), mechanical lateral distal femur angle (mLDFA), anatomical lateral distal femur angle (aLDFA), medial proximal tibial ankle (MPTA), and lateral distal tibial ankle (LDTA), with all ranging from 81° to 90°. We propose an easier screening method to identify the site of deformity of the lower limb.

**Fig. 1:**
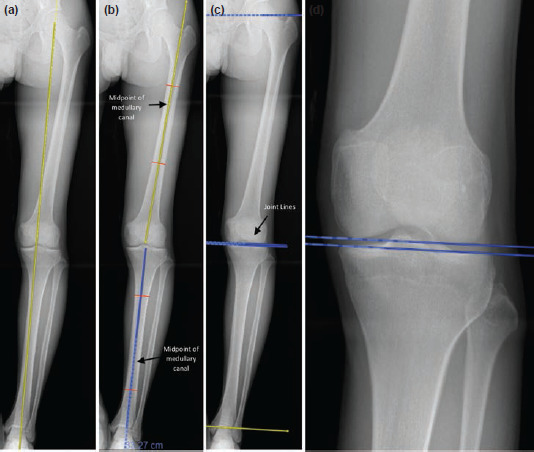
(a) Mechanical axis is drawn from the center of femoral head to the midpoint of the ankle. (b) Anatomical axis is drawn for both femur and tibia by connecting the midpoint of the medullary canal. (c, d) Modified joint line convergence angle (mJLCA) is measured by comparing four imaginary lines. The four lines include a line from the center of femoral head to the proximal part of greater tuberosity, distal femur joint line, proximal tibia joint line and ankle joint line.

There are three components in this screening technique, namely the mechanical axis, anatomical axis and the modified joint line convergence angle (Fig. 1). First, mechanical axis is drawn from the centre of femoral head to the midpoint of the ankle. Next, anatomical axis is drawn for both femur and tibia by connecting the midpoint of the medullary canal. Finally, the modified joint line convergence angle (mJLCA) is measured by comparing four imaginary lines. The four lines include a line from the centre of femoral head to the proximal part of greater tuberosity, distal femur joint line, proximal tibia joint line and ankle joint line. All four lines should be parallel to each other. Any deviation of more than 3° indicates articular deformity.

**Fig. 2:**
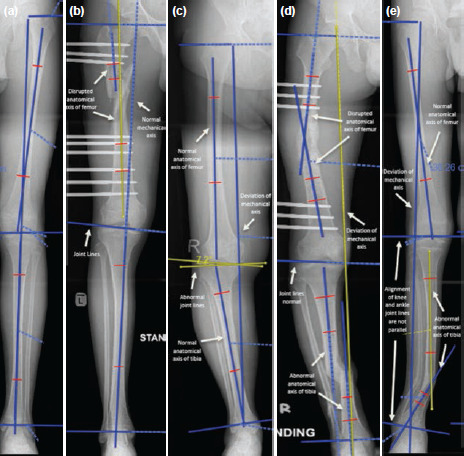
(a) Normal mechanical axis, normal anatomical axis for both tibia and femur with parallel mJLCA (all four imaginary lines are parallel to each other). In this case, the femur, tibia and joint are all normal, provided there is no shortening compared to the contralateral leg. (b) Normal mechanical axis but abnormal anatomical axis of either femur or tibia bone. The mJLCA may be normal. This indicates femur or tibia compensated deformity. (c) Abnormal mechanical axis, normal anatomical axis for both tibia and femur with disrupted mJLCA. This indicates deviated mechanical axis secondary to joint pathology. (d) Abnormal mechanical axis, abnormal anatomical axis of either femur or tibia bone with normal mJLCA. In this case, the pathological bone is causing the mechanical axis deviation. (e) The mechanical axis deviation and disrupted modified joint line convergence angle (mJLCA) is due to the tibial deformity.

There will be four potential outcomes in this screening technique:

1. Normal mechanical axis, normal anatomical axis for both tibia and femur with parallel mJLCA (all four imaginary lines are parallel to each other). In this case, the femur, tibia and joint are all normal, provided there is no shortening compared to the contralateral leg (shortening indicates sagittal deformity in the absence of coronal deformity) (Fig. 2a)
2. Normal mechanical axis but abnormal anatomical axis of either femur or tibia bone. The mJLCA may be normal. This indicates femur or tibia compensated deformity (Fig. 2b).
3. Abnormal mechanical axis, normal anatomical axis for both tibia and femur with disrupted mJLCA. This indicates deviated mechanical axis secondary to joint pathology (Fig. 2c).
4. Abnormal mechanical axis, abnormal anatomical axis of either femur or tibia bone with normal mJLCA. In this case, the pathological bone is causing the mechanical axis deviation (Fig. 2d and e).

By using this simple screening method, one can easily and swiftly locate any lower limb bone or joint pathology with only seven straight lines (mechanical axis, femur anatomical axis, tibia anatomical axis, and four parallel joint lines as mentioned above).
